# The role of gilts in transmission dynamics of swine influenza virus and impacts of vaccination strategies and quarantine management

**DOI:** 10.1186/s40813-022-00261-2

**Published:** 2022-05-05

**Authors:** Pia Ryt-Hansen, Henriette Guldberg Nielsen, Simon Smed Sørensen, Inge Larsen, Charlotte Sonne Kristensen, Lars Erik Larsen

**Affiliations:** 1 Department of Veterinary and Animal Sciences, University of Copenhagen, Grønnegårdsvej 2, 1870 Frederiksberg C, Denmark; 2 Danish Pig Research Centre, SEGES, Agro Food Park 15, 8200 Aarhus, Denmark

**Keywords:** Swine influenza A virus, Enzootic infections, Gilts, Quarantine, Biosecurity, Vaccination, Management

## Abstract

**Background:**

Along with an expanding global swine production, the commercial housing and management of swine herds, provide an optimal environment for constant circulation of swine influenza virus (swIAV), thereby challenging farmers and veterinarian in determining optimal control measures. The aim of this study was to investigate the role of gilts in the swIAV transmission dynamics, and to evaluate the impact of different control measures such as quarantine and gilt vaccination.

**Methods:**

The study was conducted as a cross-sectional study in ten Danish sow herds, including five swIAV vaccinated and five unvaccinated herds. Blood- and nasal swab samples of gilts, first parity sows and their piglets were collected at different stages in the production system (quarantine in/out, mating, gestation and farrowing) and analyzed for the presence of swIAV and swIAV antibodies. Associations between the detection of swIAV, seroprevalence, antibody levels, sow and gilt vaccination strategy and quarantine biosecurity were thereafter investigated to identify possible risk factors for swIAV introductions and persistence within the herds.

**Results:**

Nine of the ten herds of the study had swIAV circulation and swIAV was detected in the quarantine, mating- and farrowing unit. The prevalence of seropositive gilts and first parity sows was significantly higher in the vaccinated herds, but swIAV was still present in nasal swabs from both gilts, first parity sows and piglets in these herds. Quarantine gilt vaccination and all-in/all-out management resulted in a significant reduction of swIAV positive gilts at the end of the quarantine period.

**Conclusion:**

The results underline that herd vaccination and/or quarantine facilities are crucial to avoid swIAV introductions into sow herds.

**Supplementary Information:**

The online version contains supplementary material available at 10.1186/s40813-022-00261-2.

## Background

During the last 10 years, several studies have described the herd-level persistence of swIAV as a consequence of the expanding swine production favoring large-scale production systems [1–7]. These production systems, if not applying very strict biosecurity measures to avoid mixing of age groups etc., provides a constant weekly flow of naïve individuals ensuring continuous swIAV circulation [1–3, 8]. Only few studies have investigated the role of gilts and first parity sows in the within-herd persistence of swIAV [7, 9–12]. However, gilts potentially play a major role for novel swIAV introductions as the majority of the sows-herds only introduce animals from an outside source in relation to replacement of the breeding stock [7, 13]. Moreover, if the gilts are not immunized properly before entering the sow herd, they may be naïve to the circulating swIAV herd strain, thereby contributing to the continued enzootic swIAV circulation [8]. Two previous studies have shown correlations between introduction of gilts and an increase risk of herd-level persistence of swIAV [9, 10]. To the author’s knowledge, however, none of the previous studies sampled gilts and first parity sows at different stages of the production system/ages, and further associated the detection of swIAV and swIAV antibodies with the herd vaccination strategy and quarantine measures.

The aim of this study was therefore to investigate the role of the gilts in the swIAV transmission dynamics, by assessing the prevalence of swIAV and swIAV antibodies of gilts and first parity sows in the period from arrival to the quarantine unit and until 1-week after farrowing. A secondary aim was to evaluate associations between swIAV vaccination, quarantine managements and biosecurity measures in the herds on the prevalence of swIAV and swIAV antibodies.

## Methods

### Ethical statement

This study was carried out in strict accordance with the guidelines of the Good Experimental Practices (GEP) standard adopted by the European Union. In addition, all experimental procedures were conducted in accordance with the recommendations provided by University of Copenhagen, Department of Veterinary and Animal Sciences.

### Study design

In the study ten herds were selected by convenience in cooperation with the herd veterinarian. Inclusion criteria were; a herd size of minimum 800 sows to ensure enough gilts for sampling in each unit, freedom from porcine reproductive and respiratory syndrome virus (PRRSV) or PRRSV-stability (no clinical signs), presence of minimum one quarantine unit and purchase of gilts from an outside source. In addition, five of the herds should perform swIAV vaccination with Respiporc FLU3 (Ceva Santé Animale, France) and have a similar vaccination strategy including three mass sow vaccinations per year and a separate strategy for gilt vaccinations. The mass sow vaccination implies that all gilts and sows present on the farm is vaccinated at the same time. Two swIAV vaccines are available on the Danish market, with Respiporc FLU3 (Ceva Santé Animale, France) targeting the most prevalent swIAV strains circulating in Denmark [14]. The five remaining sow herds should not have performed any swIAV vaccination for at least 1 year.

**Fig. 1:**
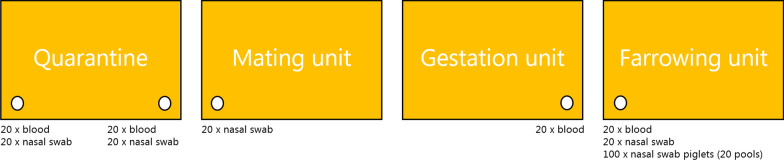
Overview of sampling in each herd. The figure illustrates the four different units/ages in which the gilts and first parity sows + piglets were sampled in each of the ten herds, and the type and number of samples obtained. The white dots indicate the approx. time at which the samples were obtained in each unit being at approx. 1 week after entry and approx. 1 week before leaving the quarantine, approx. 1 week after being transferred to the mating unit, approx. 1 week before leaving the gestation unit and after approx. 1 week after farrowing

The sampling was performed as a cross sectional study in each herd, with the aim of obtaining all samples the same day. However, in seven of the ten herds it was not possible to sample newly introduced gilts in the quarantine and gilts ready to leave the quarantine on the same day. Therefore, two separate visits were made in these seven herds. The sampling schedule is shown in Fig. 1.

A sample size of 20 gilts and 20 litters was selected for each unit due to economical and practical constraints. This sample size allowed a detection of one antibody or virus positive individual at a unit prevalence of at least 14%. In each herd, 20 gilts were blood sampled and nasal swabs were collected approximately 1 week after arrival to the quarantine, and 1 week before leaving the quarantine. In addition, 1 week after arrival to the mating unit, 20 nasal swabs were collected from gilts, whereas only blood samples were obtained from 20 gilts approx. 1 week before leaving the gestation unit. Finally, 1 week after farrowing, blood samples and nasal swabs were collected from 20 first parity sows (gilts having their first litter) and at the same time, together with nasal swabs from five piglets of the litter of each sampled sow, which were pooled in one tube. If more than 20 gilts were present in a specific batch of one of the stable units (quarantine, mating unit, gestation unit and farrowing unit) the sampling was randomized by counting the number of gilts in the batch and dividing the number with the sample size thereby obtaining the sampling frequency. If the outcome was a decimal number, it was rounded up. If there were less than 20 gilts within a batch, gilts from the previous batch (1 week younger) were included until reaching 20. The term “gilt” is used for female pigs pregnant with their first litter, whereas they are termed “first parity sows” after giving birth to their first litter.

In herd 1, additional samples were collected 3 months after the first visit to investigate if the virus identified during the first sampling had spread from the quarantine to the sow herd (farrowing unit). The additional samples included 12 pooled samples from the farrowing unit from piglets of 1 to 4 weeks of age, resulting in three pools of five nasal swabs collected from each age group.

Blood was sampled from *vena jugularis* and stored in vacutainer serum tubes (Becton–Dickinson, Denmark). Nasal swabs were collected from both nostrils with small or large sterile rayon swabs (Medical Wire, UK) and inserted into both nostrils and turned 360 degrees. The nasal swabs were preserved in a 5 mL Eppendorf tube containing 2 mL sterile 0.9% isotonic NaCl. All samples were stored at 5–8 °C until arriving at the laboratory within 12–48 h. At arrival at the laboratory, all blood samples were centrifuged at 3000 RPM for ten minutes to obtain the sera, which were subsequently frozen until further analysis at − 20 °C. Similarly, all nasal swabs were vortexed and approx. 600 μl of each sample were poured into 1.5 mL Eppendorf tube and stored at − 80 °C until further analysis.

### Analysis of blood samples

Sera were screened for antibodies against the highly conserved nucleoprotein (NP) of IAV using a commercial blocking enzyme-linked immunosorbent assay (ELISA) (IDEXX Influenza A Ab Test, IDEXX Laboratories, Inc.) following the recommended procedure. Samples with a sample-to-negative (S/N) value < 0.60 were considered positive for IAV antibodies and samples S/N ≥ 0.60 were considered negative. The individual S/N values were used as a measure of the antibody level for subsequent analysis. As the applied ELISA was a blocking ELISA a low S/N value indicated high levels of swIAV antibodies.

### Test of nasal swabs for swIAV virus by real-time RT PCR

The nasal swabs were centrifuged and 200μL were transferred to the sample rack and mixed with 400 μl RLT-buffer (QIAGEN, Copenhagen, Denmark) containing 2-mercaptoethanol (Merck, Darmstadt, Germany). Thereafter, all pathogen nucleic acids were extracted from the nasal swabs using the Cador Pathogen 96 QIAcube HT Kit (QIAGEN) automated on the Qiacube HT (QIAGEN) according to instructions from the supplier.

The resulting extractions were subjected to a previous published real-time RT PCR targeting the matrix gene of IAV to determine if the sample was swIAV positive [15]. The real-time RT PCR was run on the Rotor-Gene Q (QIAGEN) using the following program: 50 °C, 30 min; 95 °C, 15 min; cycling 45 × (95 °C, 10 s, 60 °C 20 s, 64 °C 1 s, 68 °C 1 s, 72 °C 30 s). A positive and negative control were included in all runs, and a sample was considered positive when having a Ct value < 36. The IAV positive samples had their HA and NA lineage determined using a previous published real-time RT PCR multiplex assay [1].

The two samples with the lowest Ct value from each herd were selected for Sanger sequencing of the HA and NA gene as previously described [16]. The sequencing data from LGC Genomics (Berlin, Germany) resulting from the forward and reverse primers were contiged and proof-read manually. The primer binding regions were trimmed off manually to generate consensus sequences of the HA and NA gene. For determining the subtype of the samples the consensus sequences were then checked for the closest sequence match using the function “BLAST against NCBI”, and aligned using the MUSCLE algorithm [17] along with a selection of current Danish HA and NA subtypes, which were then subjected to phylogenetic analysis using the function “create neighbor joining tree”. Thereafter, the HA and NA sequences were translated into amino acids and aligned using the MUSCLE algorithm [17] with the vaccine strains of Respiporc FLU3 (accession no. GQ161124, GQ161100 and GQ161104) [18], and investigated for the overall level of amino acid identity using the function “create pairwise comparison”. Additionally, the antigenic sites (Ca1, Ca2, Cb, Sa and Sb [19–22]) of the H1 lineage was annotated to the amino acid sequences along with the receptor binding site, to determine differences in amino acid residues in these sites.

### Questionnaire

At the herd visits the farmer was interviewed, and a questionnaire plus a checklist developed for this study was filled in based on interview and farm observations. The questionnaire concerned recruitment of gilts, the quarantine, handling of gilts in the quarantine and vaccination strategy, which was left unanswered in the five unvaccinated herds. The questionnaire included 12 closed questions (Yes/No or multiple choice) and 16 semi-open questions (i.e. quantitative variables and description of restrictions after quarantine visit) (Additional file 1: Table S2). The checklist included information on internal and external biosecurity measures (i.e. change of clothes and boots in each stable unit) and other vaccinations strategies performed in the herd along with antibiotic usage (Additional file 2: Table S3).

### Statistics

Statistics were performed in R version 4.0.4. Regression models were made using Package lme4 [23].

For investigation of association of viral shedding and antibody level to vaccination program and quarantine measures, logistic and linear models were built, all with herds as fixed effect.

For investigation of the effect of quarantine characteristics on viral shedding from gilts leaving the quarantine, a new variable was made based the presence of ‘all-in/all-out’ (AIAO) management of the quarantine.

## Results

### SwIAV antibody and swIAV prevalence in the different stable units

The results of the IAV antibody ELISA and the real-time RT PCR for IAV detection in gilts, first parity sows and piglets at the different stages of the production (quarantine in/out, mating, gestation and 1-week after farrowing) for the ten study herds are summarized in Table 1.

**Table 1:** Percentage of antibody- and virus positive gilts/sows and piglets at the different sampling times

Herd	Sow herd vaccination	Quarantine in		Quarantine out		Mating unit	Gestation unit	Farrowing unit – sows		Farrowing unit – piglets
		Ab (%)	Virus (%)	Ab (%)	Virus (%)	Virus (%)	Ab (%)	Ab (%)	Virus (%)	Virus (%)
1	No	12/20 (60)	0/20 (0)	7/20 (35)	6/20 (30)	0/20 (0)	12/20 (60)	11/20 (55)	0/20 (0)	0/20 (0)
2	No	7/20 (35)	0/20 (0)	7/20 (35)	0/20 (0)	0/20 (0)	14/20 (70)	8/20 (40)	0/20 (0)	2/20 (10)
3	No	14/20 (70)	0/20 (0)	5/20 (25)	0/20 (0)	0/20 (0)	11/20 (55)	10/20 (50)	0/20 (0)	0/20 (0)
4	No	5/20 (25)	0/20 (0)	17/20 (85)	4/20 (20)	0/20 (0)	13/20 (65)	9/20 (45)	0/20 (0)	7/20 (35)
5	No	19/20 (95)	0/20 (0)	20/20 (100)	0/20 (0)	0/20 (0)	19/20 (95)	20/20 (100)	0/20 (0)	3/20 (15)
	Total	57/100 (57)	0/100 (0)	56/100 (56)	10/100 (10)	0/100 (0)	69/100 (69)	58/100 (58)	0/100 (0)	12/100 (12)
6	Yes	3/20 (15)	10/20 (50)	5/20 (25)	3/20 (15)	1/20 (5)	20/20 (100)	20/20 (100)	0/20 (0)	0/20 (0)
7	Yes	0/20 (0)	0/20 (0)	17/20 (85)	0/20 (0)	1/20 (5)	16/20 (80)	17/20 (85)	1/20 (5)	0/20 (0)
8	Yes	14/20 (70)	0/20 (0)	20/20 (100)	8/20 (40)	1/20 (5)	20/20 (100)	20/20 (100)	0/20 (0)	1/20 (5)
9	Yes	13/20 (65)	2/20 (10)	4/20 (20)	1/20 (5)	0/20 (0)	20/20 (100)	20/20 (100)	1/20 (5)	2/20 (10)
10	Yes	11/20 (55)	0/20 (0)	20/20 (100)	1/20 (5)	1/20 (5)	16/20 (85)	18/20 (90)	2/20 (10)	9/20 (45)
	Total	41/100 (41)	12/100 (12)	66/100 (66)	13/100 (13)	4/100 (4)	92/100 (92)	95/100 (95)	4/100 (4)	12/100 (12)

“Ab” indicates the number and percentage of gilts/sows testing positive for IAV antibodies in the ELISA tests, whereas “virus” indicates the number and percentage of gilts/sows and piglets testing positive for swIAV in the real-time RT-PCR

Among the ten herds included in the study, two herds had swIAV positive gilts just after arrival in the quarantine, whereas six herds had swIAV positive gilts at the end of the quarantine. The viral status in and out of the quarantine was not significantly correlated (*p* = 0.5). Most herds (9 of 10) received seropositive gilts in the quarantine, but the prevalence varied greatly between herds, from 15 to 95% seropositive gilts. At the end of the quarantine period all herds had seropositive gilts, with a prevalence varying from 20 to 100%.

In the mating unit, a low prevalence of swIAV positive gilts was found as four herds had 5% of the gilts testing positive at this stage.

The prevalence of swIAV antibody positive gilts/first parity sows of the gestation and farrowing unit ranged from 55 to100% and 40 to100%, respectively. In addition, in the farrowing unit, three herds had first parity sows testing positive for swIAV in nasal swabs and six of the ten herds had swIAV positive 1-week old litters in the farrowing unit. More litters from swIAV positive first parity sows were virus positive compared to litters from swIAV negative gilts (50% vs. 11%, *p* = 0.048), though the number of virus positive individual sows was rather low (n = 4). For the effect of the antibody status of gilts, there was no statistical significant difference in the number of virus positive piglets from antibody positive or antibody negative gilts (12 vs. 11%, *p* = 0.4).

### Sow herd—swIAV vaccination

**Fig. 2:**
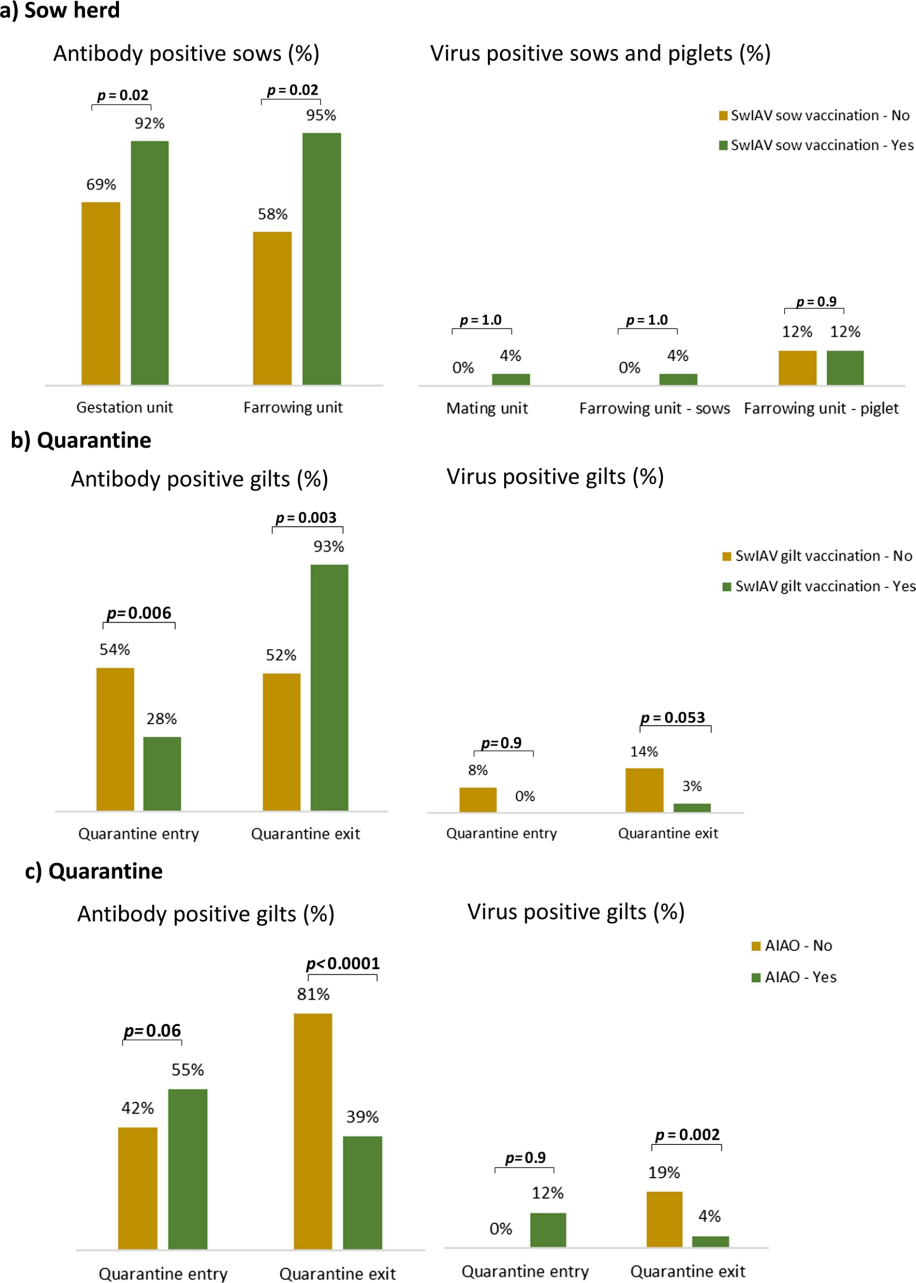
The prevalence of swIAV positive gilts/first parity sows and swIAV antibody positive gilts/first parity sows in relation to swIAV vaccination in the sow herd (**a**) and in relation to both IAV vaccination (**b**) and the presence of AIAO management (**c**) of the quarantine

The prevalence of swIAV positive gilts/first parity sows and swIAV antibody positive gilts/first parity sows in relation to the application of swIAV vaccination in the sow herd are summarized in Fig. 2.

Among non-vaccinated herds, no swIAV was found among sows in the mating and farrowing unit, however, three of five herds had swIAV circulating among piglets in the farrowing unit with a prevalence of 10 to 35% (Table 1).

Among herds with a sow vaccination strategy for swIAV, four of five herds had virus positive sows in the mating unit, all with a prevalence of 5%. In the farrowing unit, three herds had virus positive sows with a prevalence of 5 to 10% and three herds (two related to the virus positive sows) had virus positive piglets with a prevalence from 5 to 45% (Table 1).

The prevalence of virus positive sows or piglets did not differ significantly between non-vaccinated and vaccinated herds (*p* > 0.9, Table 2).

**Table 2:** IAV antibody presence, antibody level (S/N), viral presence and viral load (Ct) per stable unit for herd IAV vaccination regime, and *p* values from linear models (Antibody S/N, Virus Ct) and generalized linear models (antibody and virus positive) including herds as explanatory variable

	All herds	Herds with sow IAV vaccination		
		No	Yes	*p*
Herds, n	10	5	5	
Samples, n	200	100	100	
*Mating unit*				
Virus positive, n (%)	4 (2%)	0 (0%)	4 (4%)	1.0
Virus Ct., mean*	33.8	–	33.8	–
*Gestation unit*				
Antibody positive, n (%)	161 (81%)	69 (69%)	92 (92%)	**0.02**
Antibody S/N, mean*	0.26	0.31	0.21	**0.03**
*Farrowing unit sows*				
Antibody positive, n (%)	153 (77%)	58 (58%)	95 (95%)	**0.02**
Antibody S/N, mean*	0.25	0.33	0.20	**0.02**
Virus positive, n (%)	4 (2%)	0 (0%)	4 (4%)	1.0
Virus Ct., mean*	33.8	–	33.8	–
*Farrowing unit piglets*				
Virus positive, n (%)	24 (12%)	12 (12%)	12 (12%)	0.9
Virus Ct., mean*	27.8	26.0	29.5	0.21

Bold letters indicate a *p* value ≤ 0.05

*Among antibody or virus positive samples

The prevalence of seropositive first parity sows differed significantly between non-vaccinated and vaccinated herds in both the gestating unit (69% vs. 92%, *p* = 0.013) and farrowing unit (58% vs. 95%, *p* = 0.02). Also S/N values differed significantly between the swIAV antibody positive gilts/first parity sows of non-vaccinated and vaccinated herds in both the gestating (0.31 vs. 0.21, *p* = 0.03) and farrowing unit (0.33 vs. 0.20, *p* = 0.02), indicating significantly higher antibody levels in vaccinated gilts/first parity sows.

### Quarantine—gilt swIAV vaccination and all-in/all-out management

Among all ten herds included, two herds included a primary (prime-boost) vaccination program for gilts within the quarantine (Table 3).

**Table 3:** IAV vaccination strategies in vaccinated herds

	Herd 6	Herd 7	Herd 8	Herd 9	Herd 10
SwIAV vaccine	Respiporc FLU 3	Respiporc FLU 3	Respiporc FLU 3 + Respiporc FLUpan	Respiporc FLU 3	Respiporc FLU 3
Vaccination strategy for gilts	24 and 26 weeks-of-age	21 and 35 days after entry	26 and 28 weeks-of-age	26 and 28 weeks-of-age	2 and 23 days after entry
Primary (prime-boost) vaccination of gilts before leaving quarantine	No	Yes	No	No	Yes
Mass sow vaccination frequency per year	3	3	3	3	3
Gilts included in the mass sow vaccination	Yes	Yes	Yes	Yes	Yes
Other vaccines given at the same time	Porcilis Ery + Parvo + Lepto	Erybac Uno, Porcilis Glässer, Porcilis PCV M Hyo	Parvoruvax	Porcilis Ery + Parvo + Lepto, Porcilis Glässer	Porcilis PRRS, Porcilis PCV

A comparison of the prevalence of swIAV positive- and swIAV antibody positive gilts in relation to the application of a primary vaccination (prime-boost) program for gilts within the quarantine is summarized in Fig. 2b.

At the end of the quarantine period a significant difference was found in the prevalence of seropositive gilts (52 vs 93%, *p* = 0.003) in herds that did not vaccinate compared to herds that vaccinated gilts, and a tending difference was also found in the prevalence of virus positive gilts (14% vs. 3%, *p* = 0.053) (Table 4).

**Table 4:** IAV antibody presence, antibody level (S/N), viral presence and viral load (Ct) in quarantines with or without gilt vaccination and quarantines with or without AIAO management of the quarantine and *p* values from linear models (Antibody S/N, Virus Ct) and generalized linear models (antibody and virus positive) including herds as explanatory variable

	Total	Quarantine with gilt IAV vaccination			AIAO		
		No	Yes	*p*	No	Yes	*p*
Herds, n	10	8	2		5	5	
Samples, n	200	160	40		100	100	
*Quarantine out*							
Antibody positive, n (%)	120 (60%)	83 (52%)	37 (93%)	**0.003**	81 (81%)	39 (39%)	** < 0.0001**
Antibody S/N, mean*	0.23	0.24	0.22	0.18	0.22	0.27	0.28
Virus positive, n (%)	23 (12%)	22 (14%)	1 (3%)	0.053	19 (19%)	4 (4%)	**0.002**
Virus Ct., mean*	33.5	33.6	32.0	0.3	33.1	32.4	0.051

Bold letters indicate a *p* value ≤ 0.05

* Among antibody or virus positive samples

A comparison of the prevalence of swIAV and swIAV antibodies in relation to having all-in/all-out (AIAO) management of the quarantine is presented in Fig. 2c. Having an AIAO management of the quarantine is defined by all new pigs coming into the quarantine at the same time and all pigs leaving the quarantine at the same time resulting in no continuous intake of gilts into the sow herd. Among the ten herds, five herds had quarantines with AIAO management (Table 5).

**Table 5:** Prevalence of virus positive gilts at the end of the quarantine period per AIAO management and swIAV vaccination status

AIAO	Gilt vaccination	Herds, n	Samples, n	Virus positive, n (%)
No	No	3	60	18 (30%)
	Yes	2	40	1 (3%)
Yes	No	5	100	4 (4%)
	Yes	0	0	–
Total		10	200	23 (12%)

For herds having AIAO management a significant difference was found in the prevalence of both seropositive (81 vs. 39%, *p* < 0.0001) and swIAV positive (19 vs 4%, *p* = 0.002) gilts in the end of the quarantine period (Table 4).

Combining results for quarantine vaccination and the presence of AIAO management, five herds that neither vaccinated and nor had AIAO management of the quarantine, showed 18 (23%) virus positive gilts at the end of the quarantine period. In herds with either quarantine vaccination of gilts and no AIAO management or no vaccination of gilts and practice of AIAO management, 3% and 5% of the gilts were virus positive, respectively (Table 5). None of the ten herds had both quarantine vaccination of gilts and AIAO management.

A regression model with herds as fixed effect showed a significant lower risk of viral shedding by the end of the quarantine period with the use of swIAV vaccination or AIAO management of the quarantine (Table 6).

**Table 6:** Odds Ratios and confidence intervals from a generalized linear model for gilt SwIAV shedding (Yes/No) at the end of the quarantine with quarantine gilt vaccination, presence of AIAO management of the quarantine and herd as explanatory variables

	Model results	
	OR (CI 95%)	*p*
*Quarantine gilt vaccination*		0.003
No	–	
Yes	0.04 (0.002;22)	
*AIAO*		< 0.0001
No	–	
Yes	0.14 (0.02;0.25)	
*Herd*	1.2 (0.95;1.4)	0.06

### Questionnaires

The results of the questionnaire and the check-list are presented in Table 3 and Additional file 3: Table S1. Table 3 provides an overview of the vaccination strategies applied in the vaccinated herds. In brief, it should be noted that only two of the influenza vaccinated herds, had a primary (prime-boost) vaccination in the quarantine, and all herds included gilts/first parity sows in their mass sow vaccination program. Moreover, all vaccinated herds applied several other vaccines at the same time as the influenza vaccine. In specific, Herd 10 was applying a modified live vaccine against porcine reproductive and respiratory syndrome virus (PRRSv) at the same time as the Respiporc FLU3 vaccine (Ceva Santé Animale, France). Additional file 3: Table S1 provides information on herd size and production systems as well as health status, quarantine location and -management and IAV vaccination status of the personnel. In brief, it should be noted that five of the herds had a continuous intake of gilts into the sow herd, and that only one herd had personnel (4 out of 7 workers) that was vaccinated against human seasonal influenza virus.

### Lineages and sequencing

From six (four non-vaccinated and two vaccinated) of the nine swIAV positive herds it was possible to determine the HA and NA lineages circulating in the herds by sequencing. In the remaining three swIAV positive herds, the viral load was too low to obtain high quality sequences. The HA and NA sequences were compared to the sequences of the corresponding vaccine strains included in Respiporc FLU3 [18] (Table 3). For the four herds where an hemagglutinin (HA) protein of the Eurasian avian H1 lineage (H1av) was identified a comparison was made to the corresponding H1av vaccine component of Respiporc FLU3. The comparison revealed major differences as the overall amino acid identity ranged between 89–92%, and several residues in both antigenic sites (AS) and the receptor binding site (RBS) were divergent (Table 7 and Additional file 4: Figure S1). One of the four herds was vaccinating with Respiporc FLU3 (Herd 10). The remaining vaccinated herd wherefrom it was possible to determine the lineage of the circulating strain, it was discovered that a non-matching vaccine was applied as the H1pdm09N1av strain was detected.

**Table 7:** Identity between the H1av herd strains and the corresponding vaccine strain of Respiporc FLU3 (Ceva Santé Animale, France)

Herds	Accession number	Lineage	Identity to corresponding vaccine strain included in Respiporc FLU3	No. of mutations in AS/RBC
1		H1N1pdm09	–	
2	OM350200 OM350201	H1avN2sw	HA protein identity to HA Haselünne/IDT2617/2003: 92% NA protein identity to NA Bakum/IDT1769/2003(H3N2): 89%	AS: 5 = Cb: 2, Sa: 1, Ca: 1, Ca2: 1 and Sb: 1 and RBS: 17
4	OM350202 OM350203	H1avN2sw	HA protein identity to HA Haselünne/IDT2617/2003: 92% NA protein identity to NA Bakum/IDT1769/2003(H3N2): 89%	AS: 7 = Cb: 1, Sa: 2, Ca2: and Sb: 3 and RBS: 17
5	OM350204 OM350205	H1avN2hu	HA protein identity to HA Haselünne/IDT2617/2003: 92% NA protein identity to NA Bakum/IDT1769/2003(H3N2): 85% NA protein identity to NA Bakum/IDT1833/2000(H1N2): 82%	AS: 5 = Sa: 3, Ca1: 1, Sb:1 and RBS: 15
9	OM350207	H1pdm09N1av	– NA protein identity to NA Haselünne/IDT2617/2003: 90.4%	
10	OM350208 OM350209	H1avN2sw	HA protein identity to HA Haselünne/IDT2617/2003: 89% NA protein identity to NA Bakum/IDT1769/2003(H3N2): 88%	AS: 9 = Sa: 3, Ca1: 4, Ca2: 1 and Sb 1 and RBS: 23

The herd strains can be found in NCBI genbank, using the indicated accession numbers. “AS” indicates antigenic site, specified as Ca1, Ca2, Cb, Sa and Sb^19–22^. “RBS” indicate the receptor binding site. Numbering of amino acid are based on the first methionine (start codon). A detailed alignment are presented in Additional file 4: Figure S1

Herd 1 was a newly started herd with a new breeding stock. Interestingly, 30% of the gilts of this herd were positive for H1N1pdm09 at the end of the quarantine, thereby posing a high risk for swIAV introduction into the newly established sow herd, where no swIAV was documented at least in the gilts, first parity sows and piglets sampled in this study. It was therefore decided to investigate if swIAV were circulating within the sow herd 3 months later. Remarkably, these “follow-up” samples revealed that 9/12 pools obtained in the farrowing unit was positive for H1N1pdm09, including pigs from 1- to 4-week-of-age.

## Discussion

The results presented in this study document that gilts and first parity sows contribute to the swIAV transmission dynamics, as swIAV could be detected in these animals throughout the different stages of the production (quarantine in/out, mating and farrowing).

Depending on the different stages of the production that swIAV is detected in the gilts and first parity sows, different impact can be expected. Having swIAV virus positive gilts in the beginning of the quarantine either indicates that the virus was introduced by the gilts from the supplier herd or introduced from the sow herd into the quarantine as a consequences of inappropriate biosecurity measures [24, 25]. However, the results of this study indicate that the consequences of having swIAV present at the beginning of the quarantine does not necessarily pose a problem if the quarantine is managed with AIAO and no continuous intake of gilts, as transmission of swIAV would have stopped circulating by the end of the quarantine period. This in turn also dependent on the length of the overall quarantine period.

Having swIAV positive gilts at the end of the quarantine on the other hand, poses a major problem for the herds, as swIAV most likely will be introduced into the sow herd during the gilt-introductions. This is especially a concern if the gilts are infected with another swIAV strain/lineage than the one circulating within the sow herd, or if the sow herd is negative for swIAV, as seen in Herd 1. Fortunately, our results indicate that applying additional biosecurity measures for the gilts of the quarantine such as AIAO management can limit the presence of swIAV in the gilts before entering the sow herd. Another option is to apply a primary vaccination (prime-boost) of gilts within the quarantine, which our results also suggested to have an effect on limiting the presence of swIAV at the end of the quarantine. None of the ten included herds had both AIAO management of the quarantine and used primary swIAV vaccination (prime-boost) within the quarantine. We expect that this will be an optimal way to reduce the risk of introducing swIAV into the sow herd. The consequences of having introductions of swIAV positive gilts have been investigated in a previous study, which documented that risk having swIAV positive 3-weeks-old piglets was increased by 1.67 times, when introducing swIAV positive gilts into a sow herd [10]. These results can explain why another study found that minimizing the number of gilts introduction per year reduced the risk of enzootic level of swIAV circulation using mathematical modelling [9].

Another route of novel IAV introduction into the herds is by infected personnel. Remarkably, none of the study herds encouraged their employees to get the seasonal flu vaccination and only a few employees in one of the herds were vaccinated against IAV. In Herd 1, H1N1pdm09 was likely introduced into the quarantine from an outside source such as persons working in the herd, as the virus was only present by the end of the quarantine period. It is unlikely that the virus was introduced into the quarantine from the sow herd, since the sow herd was newly established and tested negative for swIAV. A further support of this is that the H1N1pdm09 was the dominating seasonal flu in Denmark during the sampling period [26].

SwIAV was detected in gilts of the mating unit 1 week after introduction into the sow herd, implying that gilts could have introduced the virus into the sow herd from the quarantine. Another option is that the gilts became infected in the mating unit as they entered the sow herd naïve to the herd strain. For the vaccinated herds this could be due to the gilts not receiving a complete primary (prime-boost) swIAV vaccination within the quarantine, or due to a lack of homology between the antibodies stimulated by the vaccine strains, and the herd strain. Indeed great variation was found between the HA and NA proteins of the vaccine strains the different herd strains sequenced in this study. Moreover, several of the changes identified between the vaccine strain and the herd strain were identified in antigenic sites and the receptor binding site, indicating that they could negatively impact the efficiency of the vaccine. Several previous studies have documented massive genetic drift of the H1av lineage, and have related this to major antigenic distances indicating that a lack of cross-protection between different H1 strains is highly probable [13, 14, 27–32].

Based on our results it does seem that the vaccine is capable of stimulating a pronounced antibody response, as herd applying the vaccine generally had a higher prevalence of antibody positive gilts and first parity sows. However, the results did not document that this increased prevalence of seropositive gilts and first parity sows was related to a lower incidence of swIAV detection in piglets. This could emphasize the importance of having homology between the vaccine strain and the herd strains [33, 34], as is also true for human IAV vaccines, where it is known that few mutations in antigenic sites can lead to very low efficiencies [35, 36]. However, it is also not expected that the vaccine Respiporc FLU3 (Ceva Santé Animale, France) will lower the prevalence of swIAV in the herds, as it does not claim to provide sterile immunity but reduces clinical signs and spread of virus to the lungs [18], which was not evaluated in this study. It should be mentioned that the herds included in this study was not randomly selected and therefore the selection could be biased. However, all calculations have taken into account the herd-to-herd variations.

When choosing a vaccination strategy in a given herd, the duration of immunity stimulated by the vaccine is important to consider. In general, the swIAV vaccines available in Denmark claim 3–6 months duration of immunity [18, 37]. This means that herds applying mass sow vaccination three times a year, as in this study, might have a problem with keeping a high antibody level in the sows. Moreover, by using mass sow vaccination you risk not to vaccinate the gilts/first parity sows again before their first farrowing, meaning that they lack a booster that may impair the amount of antibodies transferred to the pigslets though colostrum MDAs pre-farrowing. Previous studies have documented that the level of MDA obtained in the piglet is important for protection against swIAV infection and disease [38–41]. Therefore a separate vaccination strategy should be applied for the gilts after they enter the sow herd to secure sufficient level of MDA.

On another note, all the vaccinated herds of this study performed swIAV vaccination of their gilts at the same time as other vaccinations. Herd 10 performed vaccination against PRRSv, using a modified live vaccine (MLV). To the authors knowledge possible interactions between swIAV and PRRSv vaccines have not been investigated. However, another study has revealed that a MLV PRRSv vaccine applied together with an inactivated mycoplasma vaccine had a negative impact [42], and co-infections with PRRSv and swIAV have been shown to enhance disease severity [43]. Therefore, possible interactions should therefore be investigated between swIAV and MLV in future studies.

Finally the detection of swIAV in 1-week-old piglets confirms the results of several previous studies documenting very early swIAV infections, and further confirms that the transmission of swIAV from sow-to-piglet and/or piglet-to-sow as also described previously [1, 5, 8, 13, 16, 44]. Having swIAV circulation in the farrowing unit is not ideal as the hyper-proliferative sows are under high performance pressure and in the peak of lactation. Moreover, the piglets are often being mingled from sow to sow during this period thereby contributing to the further transmission of swIAV among the piglets and the enzootic swIAV herd status.

## Conclusion

In conclusion, the results indicate that gilts and first parity sows contribute significantly in the continuous transmission of swIAV and in novel introductions of swIAV in the herds. However, proper quarantine management including AIAO and a primary (prime-boost) swIAV vaccination in the quarantine can help reduce the presence of swIAV in gilts before they enter the sow herd. Moreover, mass sow vaccination programs can increase the prevalence of antibody positive sows and the level of antibodies, but other factors such as vaccine-herd strain homology might affect the benefit of the vaccine.

## Supplementary Information

**Additional file 1: Table S2.** Questionnaire.

**Additional file 2: Table S3.** Checklist.

**Additional file 3: Table S1.** Herd information including biosercurity measures and qurantine management. * “SPF status” is a health status that a given herd can obtain by declaring free from specific pathogens. A blue status indicates that you are free from Mycoplasma hyopneumoniae (MYC), Actinobacillus pleuropneumoniae serotype 2 (AP2), 6 (AP6) and 12 (AP12), PRRSv type 1 and 2, Brachyspira hyodysenteriae, Pasteurella multocida, Sarcoptes Scabiei var. Suis and Haematopinus suis. “NA” indicates that the herd did not have a SPF health status.

**Additional file 4: Figure S1.** Amino acid alignment of the H1av vaccine strain included in Respiporc FLU3 (Haselünne/IDT2617/2003 (H1N1)), and the four H1av strains obtained in Herd 2, Herd 4, Herd 5 and Herd 10. Amino acid differences between the vaccine strain and the herd strains are marked in red and the letter indicate the specific amino acid residue. The grey arrows highlights the antigenic sites of H1 (Ca1, Ca2, Cb, Sa and Sb), whereas the green arrow highlights the receptor binding site.

## Data Availability

All data are included in the manuscript and supplementary material. Sequences are available at NCBI Genbank.

## Abbreviations

AIAO: All in/all out
AS: Antigenic site
Ct: Cycle threshold
ELISA: Enzyme-linked immunosorbent assay
HA: Hemagglutinin
IAV: Influenza A virus
MDA: Maternally derived antibodies
MLV: Modified live vaccine
NA: Neuraminidase
PRRSv: Porcine reproductive and respiratory syndrome virus
RBS: Receptor binding site
real-time RT PCR: Real-time reverse transcription polymerase chain reaction
swIAV: Swine influenza A virus
