# Supplementary figures and images for: The role of gilts in transmission dynamics of swine influenza virus and impacts of vaccination strategies and quarantine management
Source: Porcine Health Manag. 2022 May 5;8:19. doi: 10.1186/s40813-022-00261-2 (PMC9069814; doi:10.1186/s40813-022-00261-2)

Table S2. Questionaire.


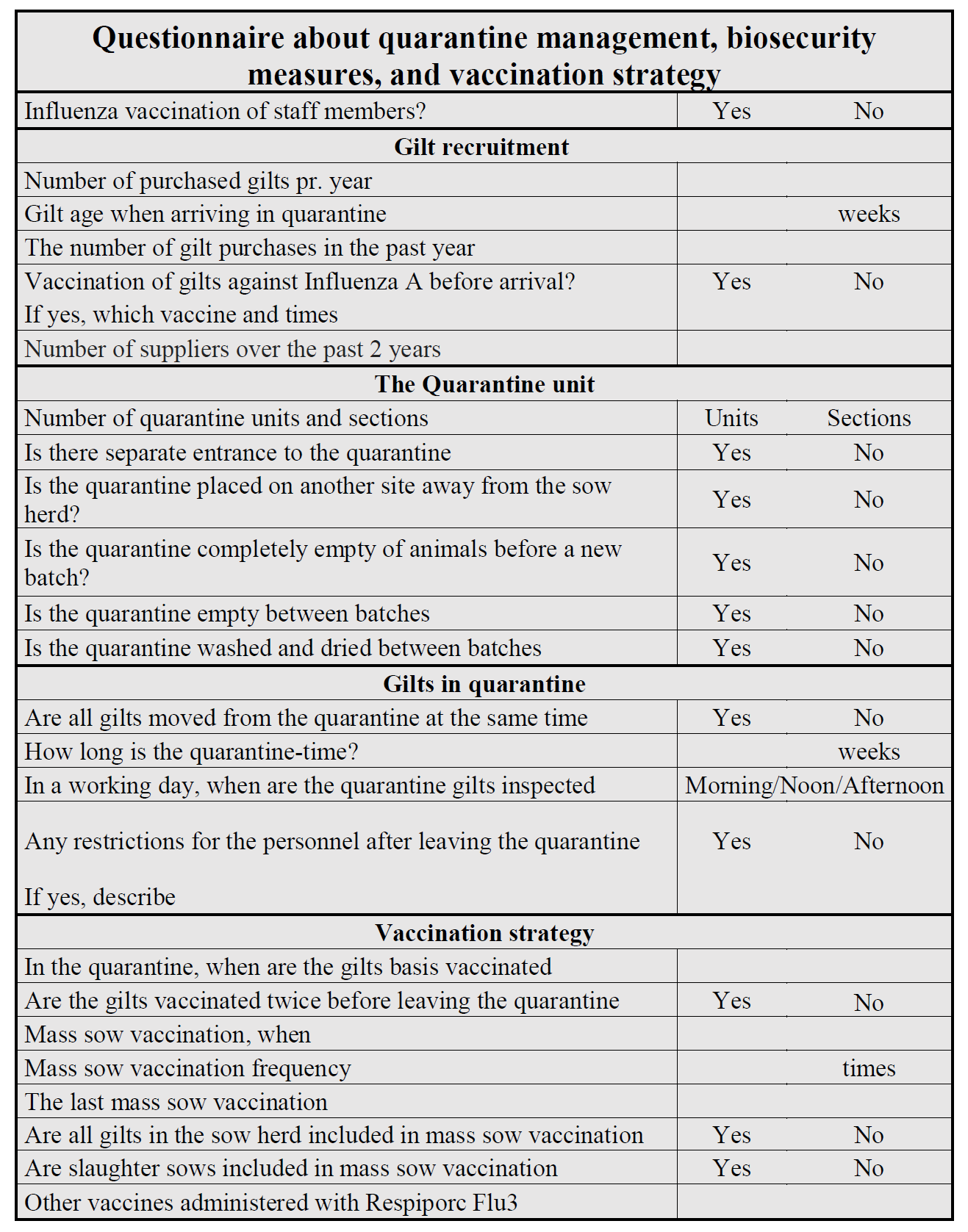

Supplement: Supplementary file 1 — Additional file 1: Table S2. Questionnaire. [file 40813_2022_261_MOESM1_ESM.docx]

Table S3. Checklist.


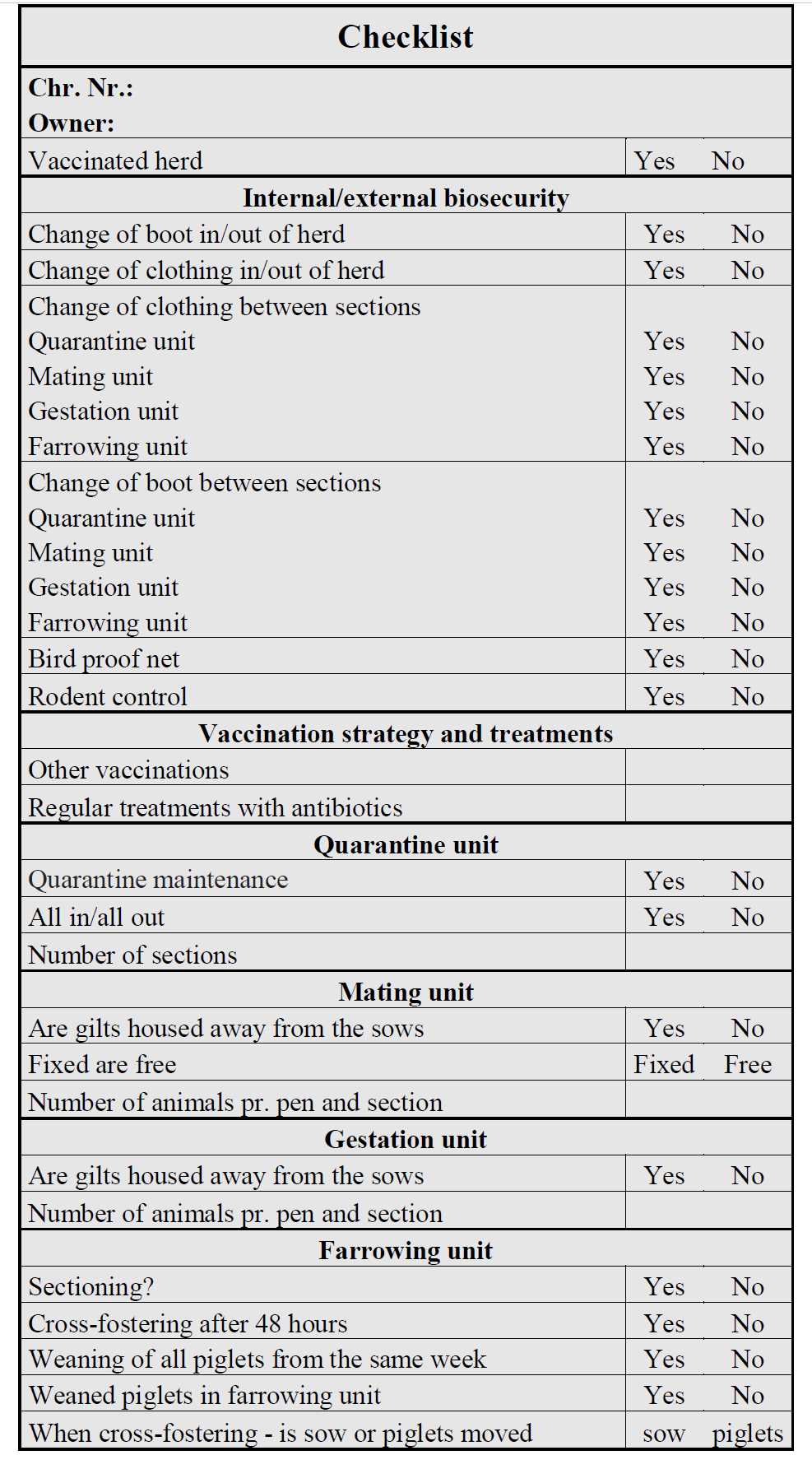

Supplement: Supplementary file 2 — Additional file 2: Table S3. Checklist. [file 40813_2022_261_MOESM2_ESM.docx]
