# Supplementary material for: The role of gilts in transmission dynamics of swine influenza virus and impacts of vaccination strategies and quarantine management
Source: Porcine Health Manag. 2022 May 5;8:19. doi: 10.1186/s40813-022-00261-2 (PMC9069814; doi:10.1186/s40813-022-00261-2)
Supplement: Supplementary file 3 — Additional file 3: Table S1. Herd information including biosercurity measures and qurantine management. * “SPF status” is a health status that a given herd can obtain by declaring free from specific pathogens. A blue status indicates that you are free from Mycoplasma hyopneumoniae (MYC), Actinobacillus pleuropneumoniae serotype 2 (AP2), 6 (AP6) and 12 (AP12), PRRSv type 1 and 2, Brachyspira hyodysenteriae, Pasteurella multocida, Sarcoptes Scabiei var. Suis and Haematopinus suis. “NA” indicates that the herd did not have a SPF health status. [file 40813_2022_261_MOESM3_ESM.docx]

Table S1. Herd information including biosercurity measures and qurantine management

|  | No. of sows | SPF health status* | Production form | IAV vaccination of personnel | No. of quarantines/sections | Quarantine location, entrance an biosecurity measures | Quarantine length | Cleaning of quarantine between batches | Intake of gilts in the herd |
| --- | --- | --- | --- | --- | --- | --- | --- | --- | --- |
| Herd 1 | 1500 | Blue | 7kg pigs | 4/7 vaccinated | 1/1 | Connected to the sow herd. Separate entrance + change of boots and clothes | 8-10 weeks | Washed and dried for 3 days | Continuous |
| Herd 2 | 1000 | Blue + MYC + AP12 | 30kg pigs | No | 1/2 | 2km from sow herd. Separate entrance + change of boots and clothes | 6 weeks | No washing but empty for 1-14 days | 4 times/year |
| Herd 3 | 1000 | Blue + AP12 | Gilts and boars | No | 1/2 | Connected to the sow herd. Separate entrance + change of boots and clothes | 8-10 weeks | Washed and dried for 3 days | 5 times/year |
| Herd 4 | 930 | Blue | 30kg pigs | No | 1/1 | Part of fattening unit. Separate entrance + change of boots and clothes + hand wash | 6-8 weeks | No washing and no empty days | Continuous |
| Herd 5 | 1000 | Blue + MYC + AP12 | 30kg pigs | No | 1/1 | 50 meters from sow herd. Separate entrance + change of boots and clothes | 6-8 weeks | No washing but empty for 1-14 days | 6-7 times/year |
| Herd 6 | 2500 | NA | 30kg pigs | No | 1/1 | 10km from the sow herd. Separate entrance + change for clothes | 8-10 weeks | Washed and dried for 3-14 days | 5 times/year |
| Herd 7 | 1900 | Blue + MYC + AP12 | Ask | No | 2/2 | 4km from sow herd but next to the nursery. Separate entrance. Change of clothes and boots + hand wash | 6 weeks | No washing but empty for 7 days | Continuous |
| Herd 8 | 860 | NA | 7kg pigs | No | 1/1 | 400m from the sow herd but next to the nursery. Separate entrance. Change of clothes but no hand washing | 8 weeks | Washing of quarantine and empty for 1-7 days | Continuous - weekly |
| Herd 9 | 1000 | Blue + MYC + AP2 + AP12 | 30kg pigs | No | 1/1 | Made as an extension to the sow herd with no separate entrance + change of clothes and boots + use of gloves | 11 weeks | Washed and dried for 5 days | 4 times/year |
| Herd 10 | 1050 | Blue + MYC + PRRS type 1 | 30kg pigs | No | 2/1 | Made as an extension to the sow herd. Separate entrance + change of clothes and boots + hand wash | 5-7 weeks | Washed and dried 5-10 days | Continuous |

*“SPF status” is a health status that a given herd can obtain by declaring free from specific pathogens. A blue status indicates that you are free from Mycoplasma hyopneumoniae (MYC), Actinobacillus pleuropneumoniae serotype 2 (AP2), 6 (AP6) and 12 (AP12), PRRSv type 1 and 2, Brachyspira hyodysenteriae, Pasteurella multocida, Sarcoptes Scabiei var. Suis and Haematopinus suis. “NA” indicates that the herd did not have a SPF health status.
