# Supplementary material for: The role of gilts in transmission dynamics of swine influenza virus and impacts of vaccination strategies and quarantine management
Source: Porcine Health Manag. 2022 May 5;8:19. doi: 10.1186/s40813-022-00261-2 (PMC9069814; doi:10.1186/s40813-022-00261-2)
Supplement: Supplementary file 4 — Additional file 4: Figure S1. Amino acid alignment of the H1av vaccine strain included in Respiporc FLU3 (Haselünne/IDT2617/2003 (H1N1)), and the four H1av strains obtained in Herd 2, Herd 4, Herd 5 and Herd 10. Amino acid differences between the vaccine strain and the herd strains are marked in red and the letter indicate the specific amino acid residue. The grey arrows highlights the antigenic sites of H1 (Ca1, Ca2, Cb, Sa and Sb), whereas the green arrow highlights the receptor binding site. [file 40813_2022_261_MOESM4_ESM.docx]

Figure S1. Amino acid alignment of the H1av vaccine strain included in Respiporc FLU3 (Haselünne/IDT2617/2003 (H1N1)), and the four H1av strains obtained in Herd 2, Herd 4, Herd 5 and Herd 10.


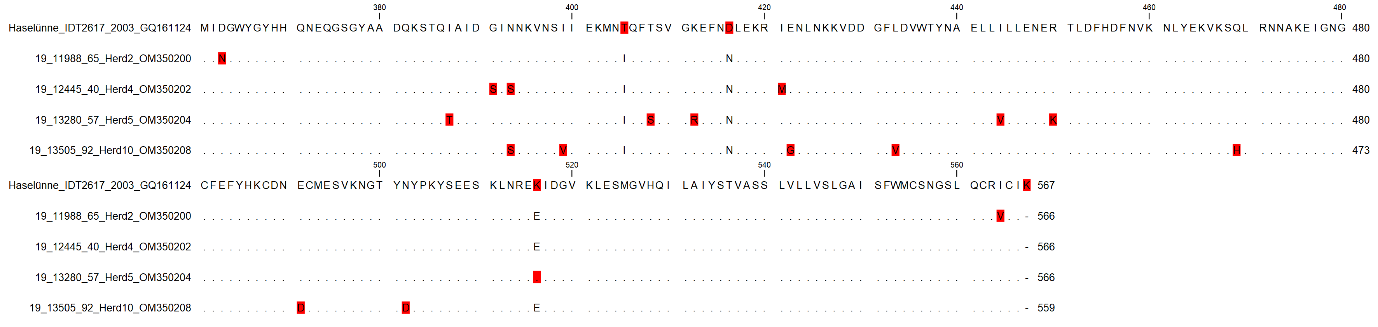

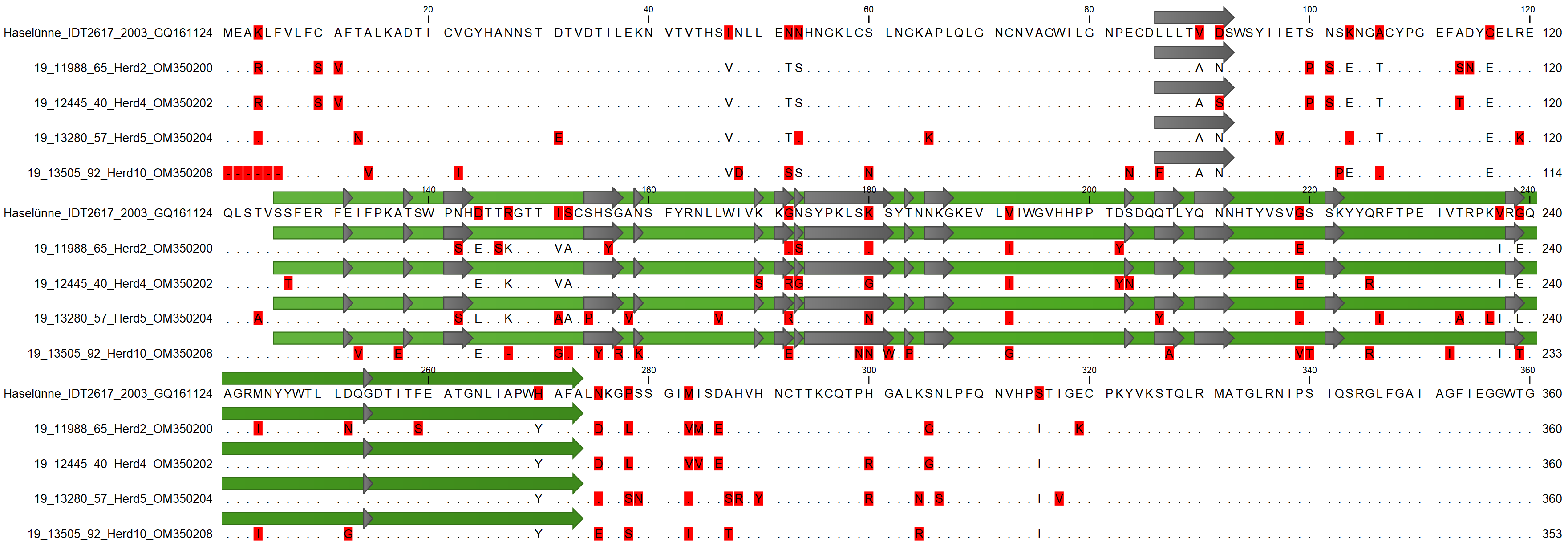


Amino acid differences between the vaccine strain and the herd strains are marked in red and the letter indicate the specific amino acid residue. The grey arrows highlights the antigenic sites of H1 (Ca1, Ca2, Cb, Sa and Sb), whereas the green arrow highlights the receptor binding site.
